# Moving Beyond Comorbidity: The Effect of Exercise Training in Community-Acquired Pneumonia

**DOI:** 10.1093/cid/ciae294

**Published:** 2024-05-31

**Authors:** Camilla Koch Ryrsø, Daniel Faurholt-Jepsen, Christian Ritz, Maria Hein Hegelund, Arnold Matovu Dungu, Bente Klarlund Pedersen, Rikke Krogh-Madsen, Birgitte Lindegaard

**Affiliations:** Department of Pulmonary and Infectious Diseases, Copenhagen University Hospital—North Zealand, Hillerød, Denmark; Centre for Physical Activity Research, Copenhagen University Hospital—Rigshospitalet, Copenhagen, Denmark; Department of Infectious Diseases, Copenhagen University Hospital, Rigshospitalet, Copenhagen, Denmark; Department of Clinical Medicine, University of Copenhagen, Copenhagen, Denmark; National Institute of Public Health, University of Southern Denmark, Copenhagen, Denmark; Department of Pulmonary and Infectious Diseases, Copenhagen University Hospital—North Zealand, Hillerød, Denmark; Department of Pulmonary and Infectious Diseases, Copenhagen University Hospital—North Zealand, Hillerød, Denmark; Centre for Physical Activity Research, Copenhagen University Hospital—Rigshospitalet, Copenhagen, Denmark; Centre for Physical Activity Research, Copenhagen University Hospital—Rigshospitalet, Copenhagen, Denmark; Department of Clinical Medicine, University of Copenhagen, Copenhagen, Denmark; Department of Infectious Diseases, Copenhagen University Hospital, Copenhagen, Denmark; Department of Pulmonary and Infectious Diseases, Copenhagen University Hospital—North Zealand, Hillerød, Denmark; Centre for Physical Activity Research, Copenhagen University Hospital—Rigshospitalet, Copenhagen, Denmark; Department of Clinical Medicine, University of Copenhagen, Copenhagen, Denmark

To the Editor—We thank Acuña-Rocha and Zuñiga-Hernandez for their interest in our study in which we investigated the effect of supervised exercise training during admission for patients with community-acquired pneumonia (CAP) on prognosis, including length of stay, risk of 90-day readmission, and 180-day mortality [1]. In the study, patients with CAP were randomly allocated to 3 parallel groups: standard care, standard care combined with daily supervised in-bed cycling, or standard care combined with daily supervised exercise according to a booklet (booklet exercise) [2]. All patients were treated for CAP according to Danish guidelines [3, 4], including ward rounds, nursing assistance, mobilization, and physiotherapy if needed.

Acuña-Rocha and Zuñiga-Hernandez raise the issue that the patients who were randomized to daily supervised exercise training had a nonstatistically significant higher percentage of chronic cardiac and respiratory diseases, which potentially could have an impact on exercise performance and the outcomes thereof. Further, there was concern about whether these patients were compensated. To ensure that no patients were pushed beyond their point of exhaustion, we reviewed patient files daily to find their individual acceptable oxygen saturation (SpO_2_), which was based on a clinical evaluation by the attending physician. Patients were given supplementary oxygen during exercise to maintain appropriate SpO_2_ (additional details provided in the Supplementary Material in Ryrsø et al [1]).

In our protocol article [5], we prespecified which covariances to include in the analysis (ie, age, sex, number of comorbidities, the confusion, urea level, respiratory rate, blood pressure, and age ≥65 years (CURB-65) score [6], and body mass index). We agree that exercise intolerance is a cardinal feature of chronic cardiac and respiratory disease [7, 8]. Hence, it would be preferable to adjust for these in the analysis. We therefore reanalyzed the study outcomes, adjusting the models for individual comorbidities that could impact exercise performance, such as heart failure, other chronic heart diseases, chronic obstructive pulmonary disease (COPD), or other chronic respiratory diseases, rather than the number of comorbidities (0, 1, ≥2). In the sensitivity analysis, supervised in-bed cycling (−5%; 95% confidence interval [CI], −26 to 22) and booklet exercise (−1%; 95% CI, −23 to 28) had no effect on length of stay compared with standard care (Table 1). Similarly, the tendency toward a reduced 90-day readmission risk with exercise remained despite adjusting for cardiac and respiratory diseases (*P* = .07). The risk of 180-day mortality was similar between standard care and the exercise groups.

**Table 1.** Percentage Decrease in Length of Stay and Risk of 90-Day Readmission and 180-Day Mortality for the In-Bed Cycling, Booklet Exercise, and Combined Exercise Training Groups, Compared With Standard Care

	Model 1		Model 2		Model 3		Model 4	
	Parameter Estimates (95% CI)	*P* Value	Parameter Estimates (95% CI)	*P* Value	Parameter Estimates (95% CI)	*P* Value	Parameter Estimates (95% CI)	*P* Value
Intention-to-treat analyses								
Length of stay								
Standard care, n = 62	Ref.		Ref.		Ref.		Ref.	
In-bed cycling, n = 61	.98 (.76–1.26)	.85	.96 (.75–1.23)	.74	.94 (.74–1.21)	.65	.95 (.74–1.22)	.69
Booklet exercise, n = 63	.99 (.77–1.28)	.96	1.00 (.78–1.27)	.99	1.00 (.78–1.28)	.98	1.00 (.77–1.28)	.97
Standard care, n = 62	Ref.		Ref.		Ref.		Ref.	
Exercise, n = 124	.98 (.79–1.23)	.89	.98 (.79–1.21)	.84	.97 (.78–1.21)	.78	.97 (.78–1.21)	.80

	Model 1		Model 2		Model 3		Model 4	
	Hazard Ratio (95% CI)	*P* Value	Hazard Ratio (95% CI)	*P* Value	Hazard Ratio (95% CI)	*P* Value	Hazard Ratio (95% CI)	*P* Value
Available-case analyses								
90-day readmission								
Standard care, n = 59	Ref.		Ref.		Ref.		…	
In-bed cycling, n = 58	.70 (.37–1.35)	.29	.65 (.34–1.25)	.20	.63 (.32–1.24)	.18	…	
Booklet exercise, n = 61	.54 (.27–1.08)	.08	.55 (.27–1.10)	.09	.52 (.25–1.07)	.08	…	
Standard care, n = 59	Ref.		Ref.		Ref.		…	
Exercise, n = 119	.62 (.35–1.09)	.10	.60 (.34–1.05)	.08	.58 (.32–1.04)	.07	…	
180-day mortality								
Standard care, n = 59	Ref.		Ref.		Ref.		…	
In-bed cycling, n = 58	1.01 (.33–3.14)	.98	.88 (.28–2.75)	.83	.81 (.25–2.60)	.72	…	
Booklet exercise, n = 61	.98 (.32–3.05)	.98	.93 (.30–2.89)	.90	.81 (.25–2.66)	.73	…	
Standard care, n = 59	Ref.		Ref.		Ref.		…	
Exercise, n = 119	1.00 (.37–2.66)	>.99	.91 (.34–2.42)	.84	.81 (.29–2.26)	.69	…	

The outcome variable length of stay was logarithm-transformed before analysis, and the regression coefficients were back-transformed to provide ratios. Data were analyzed with an analysis of covariance to quantify differences in length of stay between the standard care group and the in-bed cycling group or the booklet exercise group. The outcome variables readmission and mortality were analyzed with Cox proportional hazard regression to assess differences in time to readmission and mortality between the standard care group and the in-bed cycling and booklet exercise groups. Additional analyses were made with a combined exercise training group, which is the in-bed cycling and booklet exercise groups combined as 1 group. Model adjustments according to the different models were as follows: model 1, unadjusted model; model 2, adjusted for age and sex; model 3, adjusted for age, sex, COPD, other chronic respiratory diseases, heart failure, and other chronic heart diseases; and model 4, adjusted for age, sex, COPD, other chronic respiratory diseases, heart failure, other chronic heart diseases, the confusion, urea level, respiratory rate, blood pressure, and age ≥65 years (CURB-65) score, and body mass index.

Abbreviations: CI, confidence interval; Ref., reference.

We estimated that daily supervised exercise training during CAP admission would be more effective than standard care in reducing the length of stay. In recent years, research has been performed with the aim of improving CAP treatment, including early discharge [9, 10]. In previous studies [11, 12], early mobilization, that is, sitting out of bed for 20 minutes, was investigated as an intervention to reduce the length of stay in CAP. Unlike earlier studies, early mobilization is now a part of standard care for CAP; therefore, we investigated the additional beneficial effect of exercise training in patients with CAP. However, this trial suggests that exercise training has the potential to reduce readmission risk in patients with CAP.

## Notes

***Clinical Trials Registration***. clinicaltrials.gov: NCT04094636

***Disclaimer.*** The funders had no role in the design and conduct of the study; collection, management, analysis, and interpretation of the data; preparation, review, and approval of the manuscript; and decision to submit the manuscript for publication.

***Financial support*.** The Centre for Physical Activity Research is supported by TrygFonden (grants numbers: ID 101390, ID 20045, and ID 125132). This work was supported by grants from the Copenhagen University Hospital—North Zealand, Hillerød, Denmark (grant number: none), and the Grosserer L. F. Foghts Fond (grant number: none).

## References

[1] Ryrsø CK , Faurholt-Jepsen D, Ritz C, et al Effect of exercise training on prognosis in community-acquired pneumonia: a randomized controlled trial. Clin Infect Dis 2024; 78:1718–26. 10.1093/cid/ciae147 PMC11175663 38491965

[2] Pedersen BK , Zacho M. Syg men sund og aktiv. Bianco Luno. Available at: https://www.academicbooks.dk/da/content/syg-men-sund-og-aktiv. Accessed 22 September 2023.

[3] Danish Society of Respiratory Medicine and Danish Society of Infectious Diseases . Retningslinjer for håndtering af voksne patienter indlagt med pneumoni (2021), 2021. Available at: https://www.lungemedicin.dk/pneumoni. Accessed 8 June 2023.

[4] Metlay JP , Waterer GW, Long AC, et al Diagnosis and treatment of adults with community-acquired pneumonia. An official clinical practice guideline of the American Thoracic Society and Infectious Diseases Society of America. Am J Respir Crit Care Med 2019; 200:e45–67. 31573350 10.1164/rccm.201908-1581ST PMC6812437

[5] Ryrsø CK , Faurholt-Jepsen D, Ritz C, et al The impact of physical training on length of hospital stay and physical function in patients hospitalized with community-acquired pneumonia: protocol for a randomized controlled trial. Trials 2021; 22:571. 34454594 10.1186/s13063-021-05503-2 PMC8397876

[6] Lim WS , van der Eerden MM, Laing R, et al Defining community acquired pneumonia severity on presentation to hospital: an international derivation and validation study. Thorax 2003; 58:377–82. 12728155 10.1136/thorax.58.5.377 PMC1746657

[7] Vestbo J , Hurd SS, Agustí AG, et al Global strategy for the diagnosis, management, and prevention of chronic obstructive pulmonary disease. Am J Respir Crit Care Med 2013; 187:347–65. 22878278 10.1164/rccm.201204-0596PP

[8] Vogiatzis I , Zakynthinos S. The physiological basis of rehabilitation in chronic heart and lung disease. J Appl Physiol (1985) 2013; 115:16–21. 23620491 10.1152/japplphysiol.00195.2013

[9] Fally M , von Plessen C, Anhøj J, et al Improved treatment of community-acquired pneumonia through tailored interventions: results from a controlled, multicentre quality improvement project. PLoS One 2020; 15:e0234308. 32525882 10.1371/journal.pone.0234308 PMC7289425

[10] Fally M , Israelsen S, Benfield T, Tarp B, Ravn P. Time to antibiotic administration and patient outcomes in community-acquired pneumonia: results from a prospective cohort study. Clin Microbiol Infect 2021; 27:406–12. 32896655 10.1016/j.cmi.2020.08.037

[11] Mundy LM , Leet TL, Darst K, Schnitzler MA, Dunagan WC. Early mobilization of patients hospitalized with community-acquired pneumonia. Chest 2003; 124:883–9. 12970012 10.1378/chest.124.3.883

[12] Carratalà J , Garcia-Vidal C, Ortega L, et al Effect of a 3-step critical pathway to reduce duration of intravenous antibiotic therapy and length of stay in community-acquired pneumonia: a randomized controlled trial. Arch Intern Med 2012; 172:922–8. 22732747 10.1001/archinternmed.2012.1690

